# Ethical Horizons in Robotic Rehabilitation: Ensuring Safe AI Use Under the EU AI Act

**DOI:** 10.3390/medsci13040317

**Published:** 2025-12-14

**Authors:** Rocco Salvatore Calabrò

**Affiliations:** IRCCS Centro Neurolesi “Bonino-Pulejo”, 98123 Messina, Italy; roccos.calabro@irccsme.it:

Artificial intelligence (AI) is reshaping robotic rehabilitation and shifting practice beyond pre-programmed repetitive movement patterns toward data-driven and personalised therapeutic interventions for people with neurological and musculoskeletal impairments [1,2]. Contemporary AI-enabled robotic platforms, including end-effector gait trainers, overground exoskeletons and upper-limb workstations, integrate models that continuously adjust assistance, trajectories and feedback in response to performance, physiology and context, with the promise of improving recovery while easing pressure on overstretched rehabilitation services [1,2]. Wearable robots for gait rehabilitation rely increasingly on intention-detection algorithms that infer user goals from kinematic patterns, surface electromyography or inertial sensor streams, and these systems modulate assistance in real time to maintain an assist-as-needed paradigm that aims to maximise active participation [3,4]. High-performance wearable robots that follow human-in-the-loop optimisation further blur boundaries between assistive and restorative technologies and promise to reconstruct motor function and augment sensory feedback [5].

Evidence from narrative reviews, systematic reviews and controlled trials indicates that robot-assisted gait training can enhance walking speed, endurance and functional ambulation in stroke survivors compared with conventional therapy alone, particularly when integrated into intensive multidisciplinary programmes [1,2,6,7]. Similar benefits emerge for exoskeleton-assisted walking in people with spinal cord injury and for AI-assisted rehabilitation in musculoskeletal disorders, supporting gains in pulmonary function, pain, range of motion and walking parameters within unified therapeutic frameworks [8,9,10]. Clinical heterogeneity remains a persistent feature and effect sizes vary across devices, protocols and patient subgroups, so AI-driven robotic rehabilitation cannot be treated as a universal panacea and demands rigorous evaluation [1,6,7,9]. Technological developments of this kind illustrate both the transformative potential and the ethical fragility of AI in rehabilitation, because robots that become more autonomous, more intimately coupled to the body and more deeply embedded in care pathways expose patients to amplified risks of opaque decision making, malfunction, bias and misuse. The European Union Artificial Intelligence Act (AI Act), in force since 2024, represents a landmark regulatory response to these concerns through a harmonised risk-based framework for AI systems across sectors [11]. Healthcare applications, including AI systems embedded in robotic rehabilitation devices, fall into the high-risk category because they directly affect patient safety and fundamental rights and therefore face stringent obligations [11,12].

High-risk status requires providers to implement comprehensive risk management, rely on high-quality and representative data, undergo ex ante conformity assessment, maintain post-market monitoring and secure meaningful human oversight throughout the lifecycle of the system [11,12,13,14]. Clinicians and developers can view these requirements as codified expressions of core bioethical principles that demand concrete technical and organisational implementation. Safety represents the foundational concern whenever AI assumes partial control of electromechanical systems that move fragile bodies during high-intensity training sessions. Real-time controllers that misinterpret noise as human intention or fail to detect loss of balance may cause falls, joint overstress or other adverse events that undermine the basic rehabilitative purpose of these devices [3,4,5]. The AI Act focus on continuous risk management, incident reporting and post-market surveillance aligns with a safety culture that already characterises rehabilitation practice, yet operationalising algorithmic vigilance at the bedside remains challenging [11,12,13,14]. Robust safety culture in AI-enabled robotic rehabilitation requires continuous logging of system behaviour, structured analysis of adverse events and near misses and feedback loops between clinicians, engineers and manufacturers so that usability problems rapidly inform updates to algorithms and protocols. High-risk AI systems in rehabilitation therefore belong inside learning health systems that interrogate not only clinical outcomes but also the behaviour of the AI itself. Human oversight closely follows safety as a core requirement for responsible AI-enabled rehabilitation. Robots that implement advanced control strategies can support clinical decision making but must not displace clinician responsibility for therapeutic choices. The AI Act demands design features that enable human operators to understand the role of the system, recognise erroneous outputs and intervene or interrupt operation when necessary [11,12]. Rehabilitation therapists therefore need at least conceptual insight into how assistance parameters are adapted, which triggers drive major changes in support or task difficulty and how the system responds to unusual events such as sudden increases in spasticity or fatigue. International guidelines for trustworthy medical AI highlight human-centred design, explicit allocation of clinical responsibility and training for professionals who deploy AI-enabled tools [11,12,13,14]. Meaningful oversight depends on digital literacy among rehabilitation staff, transparent documentation of algorithms and failure modes and user interfaces that highlight clinically relevant information, because otherwise human operators risk becoming passive monitors of systems they cannot properly control.

Data governance forms a second major ethical axis where robotic rehabilitation intersects with the AI Act. Robotic devices function as mobile data platforms and continuously capture movement trajectories, force profiles and physiological signals, producing longitudinal datasets that support personalisation, monitoring, algorithm retraining, secondary research and commercial development [2,3,4,9]. These datasets are powerful instruments for clinical and scientific progress and also constitute highly identifying and sensitive records, since de-identified gait signatures or muscle activation patterns may permit reidentification when linked with other sources. The AI Act and the General Data Protection Regulation mandate data minimisation, privacy by design, strong cybersecurity and clear specification of purposes for which data are processed, and these legal norms closely reflect long-standing ethical concerns about informational harm and loss of control [11,13,15]. Analyses of healthcare data mining warn that secondary use of clinical data without robust governance can erode trust and expose patients to discrimination or exploitation [15,16,17,18]. Responsible data governance for robotic rehabilitation therefore combines consent and communication about data flows with technical strategies such as federated learning and privacy-preserving analytics that support collaboration and bias mitigation.

Algorithmic bias and fairness emerge as particularly insidious ethical challenges because unfair models can silently undermine rehabilitation as a vehicle for equity and inclusion. Studies of AI in medicine document how models reproduce disparities when they learn from skewed datasets, rely on biased proxies for health or encode structural inequities in subtle patterns [18,19]. Datasets used to train controllers and decision support algorithms for robotic rehabilitation often arise from small samples in specialised centres and typically overrepresent younger and well-resourced patients with higher baseline function [1,3,6]. Device design itself frequently reflects the anthropometrics and movement patterns of average-sized adults and models calibrated on these bodies may perform poorly in older adults, women and people with atypical morphology or severe multimorbidity. Allocation of robotic rehabilitation, titration of training intensity and rules for reducing assistance may consequently disfavour people who diverge from the majority profile, including patients with cognitive deficits or limited prior technology use. Fairness-focused reviews argue that mitigation of these risks requires curated and diverse training cohorts, bias-aware performance evaluation across clinically relevant subgroups and algorithmic strategies such as reweighting, fairness constraints or robust optimisation [18,19]. The AI Act requirement that datasets for high-risk systems be relevant, representative, free of errors and complete offers a legal basis for demanding evidence of equitable performance and for conformity assessments that examine distributional as well as average effects [11,12,13]. Transparency and explainability, long recognised as ethical desiderata in medical AI, present distinctive challenges in robotic rehabilitation because controllers for exoskeletons and gait trainers often rely on layered architectures that combine low-level feedback with higher-level predictive modules, and these designs obscure the causal chain from sensor input to motor output [4,5].

Rehabilitation remains a domain where patient agency and understanding carry particular weight and individuals who undertake arduous therapy programs reasonably expect to know what they will be undergoing and why. Mapping reviews and scoping reviews on AI ethics and explainability show that different stakeholders require different styles of transparency, with clinicians needing interpretable performance metrics and documentation of failure modes and patients benefiting more from plain-language explanations of system purpose, limitations and circumstances in which clinicians might override recommendations [16,17,20]. Informed consent for AI-mediated robotic rehabilitation must reflect these insights and describe the role of AI in adjusting assistance and task parameters, acknowledge that no algorithm is infallible and outline available safeguards, including human oversight and emergency stop mechanisms. Patients who live with cognitive impairment, aphasia or limited digital literacy may require accessible multimodal communication, involvement of family or legally authorised representatives and opportunities to revisit decisions over time, because such approaches better respect autonomy than single, dense consent forms that are quickly forgotten.

Ethical horizons for AI-enabled robotic rehabilitation extend beyond compliance with the AI Act. Regulatory provisions define high-risk categories and essential requirements but do not replace professional and moral responsibilities of clinicians, researchers and manufacturers. Conceptual frameworks in bioethics and digital health argue that ethical AI in healthcare represents a continuous process of reflection and negotiation rather than static satisfaction of technical checklists [16,17]. That process begins with problem formulation, including choices about which patient groups receive priority, which outcomes are optimised and how individual benefit is balanced against costs at a system level. Participatory and co-design approaches that involve patients, caregivers, rehabilitation professionals, engineers and ethicists from early stages of development entail negotiating values and aligning system functions with real-world needs and preferences instead of abstract engineering goals [2,3,4]. Evaluation practices must also evolve because traditional clinical trials, though indispensable for establishing safety and efficacy, seldom capture the ethical and social impacts of AI-enabled robotics. Outcomes such as therapeutic alliance, perceived autonomy, trust in technology and stigma associated with visible assistive devices often influence adherence and long-term benefit yet rarely appear as primary endpoints. Systematic reviews report inconsistent documentation of adverse events, usability issues and discontinuation reasons in robotic rehabilitation trials and this inconsistency obscures how AI-related failures manifest in real practice [6,7,9]. Qualitative methods, patient-reported outcomes and extended follow-up can generate richer accounts of lived experience with robotic systems and linking these approaches to AI Act post-market monitoring requirements supports informed decisions about updating or withdrawing technologies when unacceptable patterns arise [11,12,13,14].

Global and regional dimensions of AI governance shape the future of robotic rehabilitation. Technologies interact with reimbursement schemes, workforce shortages, infrastructural constraints and cultural attitudes toward disability and technology, and comparative analyses of regulation reveal substantial variation in how jurisdictions balance innovation and protection [12,13,14]. The EU AI Act stands out as a stringent attempt to foreground fundamental rights and safety in AI deployment and this stance creates both challenges and opportunities for manufacturers and healthcare organisations that operate across borders. Compliance with high-risk requirements may impose significant costs, particularly for small and medium-sized enterprises that develop innovative rehabilitation devices, yet alignment with this framework can function as a mark of trustworthiness and facilitate broader diffusion, echoing the Brussels effect previously observed with the General Data Protection Regulation [11,12,13,14]. The rehabilitation community now possesses an opportunity to contribute proactively to AI Act interpretation and implementation so that guidance documents, conformity assessment practices and technical standards reflect specific features of robotic rehabilitation rather than generic assumptions about software-only medical AI. AI-enabled robotic rehabilitation consequently stands at a pivotal ethical juncture. Technologies that allow robots to infer movement intention, adapt assistance and learn from large cohorts can accelerate recovery and extend access, yet can also introduce opacity, bias and new vulnerabilities into complex care pathways [1,2,3,4,5,18,19].

The EU AI Act supplies a timely and robust legal scaffold for managing these risks by classifying rehabilitation AI as high-risk and imposing demands for safety, data governance, transparency and human oversight [11,12,13,14]. Genuine ethical alignment requires more than regulatory compliance and development and the deployment of robotic rehabilitation should embed ethics throughout the lifecycle of AI systems, including participatory design, representative data collection, transparent implementation, clinician education and reflexive post-market monitoring. Collaborative engagement by clinicians, engineers, ethicists, regulators and patients can enable robotic rehabilitation to evolve not only as a powerful tool to restore movement but also as a paradigm of human-centred and rights-respecting AI in clinical practice.
